# Prevalence and risk factors of high blood pressure among persons assessed for TB at three health facilities in Lusaka, Zambia

**DOI:** 10.3389/ftubr.2025.1548843

**Published:** 2025-07-09

**Authors:** Seke G. Y. Muzazu, Daniel Siameka, Nsala Sanjase, Brian Shuma, Mary Kagujje, Monde Muyoyeta

**Affiliations:** TB Department, Centre for Infectious Disease Research in Zambia (CIDRZ), Lusaka, Zambia

**Keywords:** tuberculosis, hypertension, raised blood pressure, integrated service delivery, non-communicable diseases

## Abstract

**Background:**

Low- and middle-income countries (LMICs) are experiencing a rapidly increasing burden of non-communicable diseases (NCDs), additionally straining health systems battling with high prevalence of infectious diseases such as tuberculosis (TB). This study set out to describe the prevalence of a high blood pressure (HBP) and identify factors associated with high BP among persons with presumed and diagnosed TB patients in a high TB burden setting.

**Methods:**

We conducted secondary analysis of data from a cross-sectional study that enrolled consecutive persons with presumed TB and those at high risk for TB, aged ≥18 years old, between November 2021 and December 2022 in Lusaka, Zambia. We defined a high blood pressure (HBP) reading as any systolic ≥140 mmHg and/or diastolic ≥90 mmHg. Descriptive statistics were employed to summarize participant demographic and clinical characteristics while logistical regression analysis was used to identify factors associated with HBP.

**Results:**

Of 2,431 participants, 541 (26.9%) had HBP and 290 (11.9%) had bacteriologically confirmed TB. Among those with HBP, 51/541 (7.8%) had TB. Overall, 1,305 (53.6%) were female, median age was 35 years (IQR = 27–45), 899 (36.9%) were living with HIV, 383 (15.7%) were overweight, and 209 (8.6%) were obese. We noted a history of tobacco use among 718 (29.5%), and 1,078 (44.3%) reported alcohol use during the past year. The odds of a high BP increased with ages 35–44 (aOR = 1.57, 95%CI = 1.22–2.02); 45–54 (aOR = 2.10, 95%CI = 1.59–2.76); and over 55 (aOR = 4.30, 95%CI = 3.11–5.95) years, alcohol use (aOR = 1.40, 95%CI = 1.15–1.71), and BMI over 30 (aOR = 1.64, 95%CI = 1.20–2.25). Conversely, participants with bacteriologically confirmed TB (aOR = 0.63, 95%CI = 0.45–0.88), aged 18–24 years (aOR = 0.58, 95%CI = 0.41–0.82), and BMI <18.5 (aOR = 0.62, 95%CI = 0.47–0.80) had lower odds of HBP.

**Conclusion:**

Over 1 in 4 persons assessed for TB had HBP on initial reading. Higher BMI, alcohol use and older age were associated with increased HBP prevalence. These results provide valuable baseline data to inform strategies for integrated TB and non-communicable diseases (NCDs) including HBP screening in similar settings.

## Introduction

Hypertension is an important contributor to global morbidity and mortality (1). The World Health Organization (WHO) estimates that 1.28 billion adults aged 30–79 years are hypertensive with current data showing that only 42% of hypertensives are diagnosed and treated. Most hypertensive adults remain unaware that they have the condition while only 1 in 5 adults with hypertension have it under control (2).

In 2013, Africa was reported to have the highest population of hypertension patients globally (3) but more recent data from the WHO global report of hypertension 2023 noted that it has been surpassed by the Western Pacific and South-East Asia WHO regions (4). Unlike in the past, hypertension is now more prevalent in the low- and middle-income countries (LMICs) (5–7). Additionally, people from these countries have higher risk of death from hypertension-related complications than their counterparts in higher income countries which have been associated to lack of access to adequate healthcare services and dietary variations (6–8). LMICs are experiencing a rapidly increasing burden of non-communicable diseases (9, 10). Many of these countries also have a high burden of infectious diseases such as tuberculosis and remain largely unprepared to handle this double burden (11–13).

The prevalence of hypertension among TB patients is variable from setting-to-setting and studies typically report a single blood pressure (BP) reading (14). Hypertension is also associated with increased mortality among TB patients (15). We report on the burden of high blood pressure (HBP) and factors associated with high BP among persons being assessed for TB.

## Materials and methods

We conducted a secondary analysis of data collected from a cross-sectional study evaluating a computer-aided chest x-ray diagnostic as a screening tool for TB, among persons attending three primary health care (PHC) facilities in Zambia between October 2021 and February 2023 (16). The primary study enrolled consecutive persons who were aged ≥18 years old and had a known HIV status or willing to get tested. Participants were included if they presented with any of the standard TB symptoms; or were a TB contact; were newly diagnosed with HIV or were within 1 month of starting antiretroviral therapy (ART) regardless of symptoms. Included participants presenting with TB suggestive symptoms were defined as *presumed TB* while the rest fell in the at-risk group.

Upon enrolment into the primary study, participants had their anthropometrics and vital signs measured which included blood pressure (BP), temperature, pulse rate, weight, and height. Participants all had at least one baseline BP reading done at study enrolment with repeat BP measurements done after 15 to 30 min for participants who had a high initial reading and the lower of the two was recorded. We defined a high BP reading as any systolic ≥140 mmHg and/or diastolic ≥90 mmHg (17). In addition to a detailed clinical history and physical examination, we also collected sociodemographic characteristics at baseline which included age, sex, HIV status, prior TB, prior COVID-19, tobacco use, history of hypertension, and alcohol use. Those identified to have high blood pressure were managed appropriately by study clinicians and linked to routine services for continued care and follow-up at the health facility.

The data was analyzed using STATA Statistical Software (Stata Corporation Version 18.5 College Station, Texas 77845, USA). Descriptive statistics were reported using frequencies and percentage for categorical variables. For continuous variables that did not follow a normal distribution, the median and interquartile range (IQR) were reported. Logistic regression analysis was done to identify factors associated with high BP readings among persons assessed for and among those diagnosed with TB, as well as to identify factors associated with treatment outcomes in the same patient population. Univariable analysis was initially conducted to identify factors or variables associated with high BP reading and patient treatment outcomes. Following this, multivariable analysis was conducted to control for confounding variables. A statistical cutoff point of 0.2 (20%) was used to select variables to include in the multivariable analysis. Then in the multivariable regression analysis, the variables with the highest *p-value* (>0.05) were removed until we remained with significant variables in the model. Results were then charted onto a forest plot highlighting variables that were significant.

Ethical clearance for the primary study was obtained from the University of Zambia Biomedical Research Ethics Committee (UNZABREC) with study number 1989–2021.

## Results

### Flow of study participants

**Figure 1:**
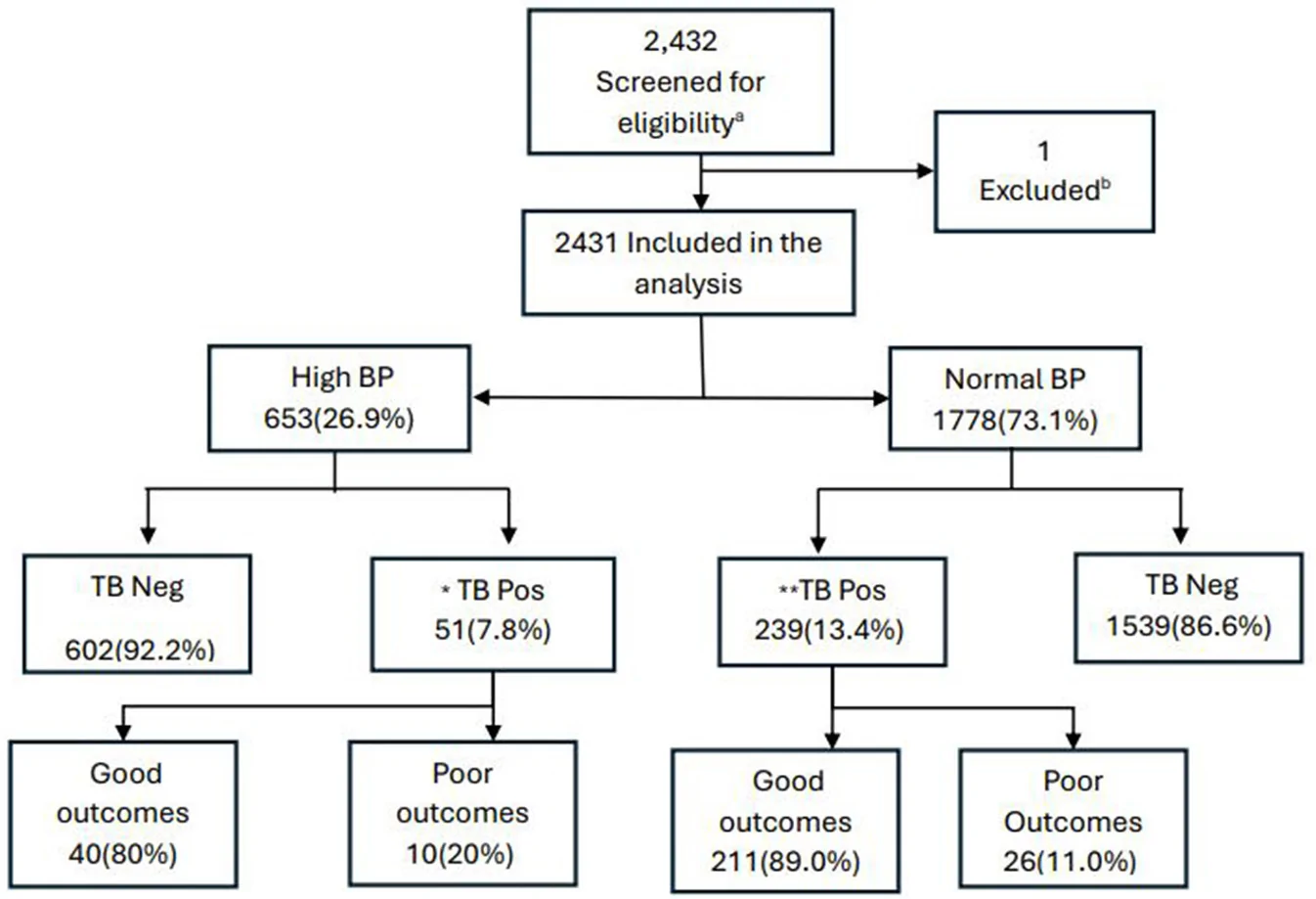
Participant flow diagram. BP, blood pressure; TB, tuberculosis; Neg, negative; Pos, positive; TB pos, bacteriologically confirmed TB. Good outcomes – cure or treatment completed; Poor outcomes – died, loss to follow-up, and treatment failed. ^a^Screened adults seeking TB services. ^b^1 participant was not eligible based on the age criteria. *1 participant had a missing outcome or not evaluated; **2 participants had missing outcomes.

Between October 2021 and February 2023, 2,432 participants were screened and we excluded those who were < 18 years old, had no TB symptoms, were not TB household contacts or not HIV newly diagnosed/ < 1 month on ART (Figure 1). Out of 2,431 included in the study analysis, 26.9% had high blood pressure (HBP) while 73.1% had a normal BP reading.

### Participant characteristics and prevalence of high blood pressure

The median age was 35 years (IQR = 27–45), females at 53.2%, HIV negative status 62.7%, 82.3% had no prior history of TB, and 91.5% had no history of COVID-19 infection. The median BMI was 21 (IQR 19.0–24.0) and the majority reported never having used tobacco (69.9%) or consumed alcohol (55.2%) in the past year. Furthermore, the prevalence of bacteriologically confirmed TB in our population was 11.9%, while that of high blood pressure (HBP) was 26.9%. Univariable analysis showed that age, priori TB, BMI, alcohol use, and bacteriologically confirmed TB were factors associated with high BP readings (Table 1).

**Table 1:** High BP reading and univariable estimates among persons assessed for TB by participants characteristics (*N* = 2,431).

**Characteristics**	**Overall**	**Blood pressure status**		**Univariable estimates**	
	**All**	**Normal (**<**140/90)** ***n*** = **1,778 (73.1%)**	**High (**≥**140/**≥**90)** ***n*** = **653 (26.9%)**	**COR (95% CI)**	***p*** **-** ***value***
	***N** =* **2,341(%)**	***n*** **(%)**	***n*** **(%)**		
**Group Age group (years)** ^a^					
Median (IQR)	35 (27–45)	33 (26–43)	41 (32–50)		
18–24	438 (18.0)	386 (21.7)	52 (8.0)	0.52 (0.37–0.73)	0.001^*^
25–34	722 (29.7)	573 (32.2)	149 (22.9)	Ref.	Ref.
35–44	635 (26.1)	448 (25.2)	187 (28.7)	1.61 (1.42–2.06)	0.001^*^
45–54	407 (16.8)	262 (14.7)	145 (22.3)	2.13 (1.62–2.79)	0.001^*^
>55	227 (9.4)	109 (6.1)	118 (18.1)	4.16 (3.03–5.71)	0.001^*^
**Sex**					
Male	1,126 (46.3)	829 (46.6)	297 (45.5)	0.96 (0.80–1.14)	0.616
Female	1,305 (53.7)	949 (53.4)	356 (54.5)	Ref.	Ref.
**Group Patient type**					
Presumptive	2,347 (96.5)	1,718 (96.6)	629 (96.3)	Ref.	Ref.
Household contacts	10 (0.4)	10 (0.6)	0 (0.0)	–	–
HIV newly diagnosed/ < 1 month on ART	74 (3.0)	50 (2.8)	24 (3.7)	1.3 (0.80–2.15)	0.284
**HIV status** ^b^					
Negative	1,524 (62.9)	1,107 (62.4)	417 (64.4)	Ref.	Ref.
Positive	899 (37.1)	668 (37.6)	231 (35.7)	0.92 (0.76–1.12)	0.371
**Group Prior TB** ^c^					
No	2,001 (82.6)	1,482 (83.5)	519 (80.1)	Ref.	Ref.
Yes	421 (17.4)	292 (16.5)	129 (19.9)	1.26 (1.00–1.59)	0.048^*^
**Group Prior COVID-19** ^c^					
No	2,225 (91.9)	1,623 (91.5)	602 (92.9)	Ref.	Ref.
Yes	70 (2.9)	48 (2.7)	22 (3.4)	1.24 (0.74–2.06)	0.416
I don't know	127 (5.2)	103 (5.8)	24 (3.7)	0.63 (0.40–0.99)	0.045^*^
**Group BMI** ^d^					
Median (IQR)	21 (19–24)	21 (19–24)	22 (20–26)		
< 18.5	542 (22.3)	444 (25.0)	98 (15.1)	0.62 (0.48–0.80)	0.001^*^
18.5–24.9	1,292 (53.3)	954 (53.7)	338 (52.2)	Ref.	Ref.
25.0–29.9	383 (15.8)	256 (14.4)	127 (19.6)	1.40 (1.09–1.79)	0.007^*^
>30.0	209 (8.6)	124 (7.0)	85 (13.1)	1.93 (1.43–2.62)	0.001^*^
**Group Tobacco use** ^e^					
Never	1,700 (70.3)	1,243 (70.2)	457 (70.5)	Ref.	Ref.
Current	405 (16.7)	291 (16.4)	114 (17.6)	1.07 (0.8–1.36)	0.607
Stopped	313 (12.9)	237 (13.4)	76 (11.7)	0.87 (0.66–1.15)	0.338
Not disclosed	2 (0.1)	1 (0.1)	1 (0.2)	2.72 (0.17–43.57)	0.480
**Group Alcohol use in past year** ^e^					
No	1,342 (55.5)	1,007 (56.8)	335 (51.7)	Ref.	Ref.
Yes	1,078 (44.3)	765 (43.2)	313 (48.3)	1.23 (1.03–1.47)	0.025^*^
**Group Bact. confirmed TB**					
Negative	2,141 (88.1)	1,539 (86.6)	602 (92.2)	Ref.	Ref.
Positive	290 (11.9)	239 (13.4)	51 (7.8)	0.55 (0.40–0.75)	0.001^*^

TB, tuberculosis; Bact, Bacteriologically; BMI, Body Mass Index; ^*^, Significant p-value, >, greater than; <, less than; COR, Crude odds ratio; CI, Confidence interval.

a n = 2 missing values,

b n = 8 missing values,

c n = 9 missing values,

d n = 5 missing values,

e n = 11 missing values.

### Factors associated with high blood pressure among persons assessed for TB

**Figure 2:**
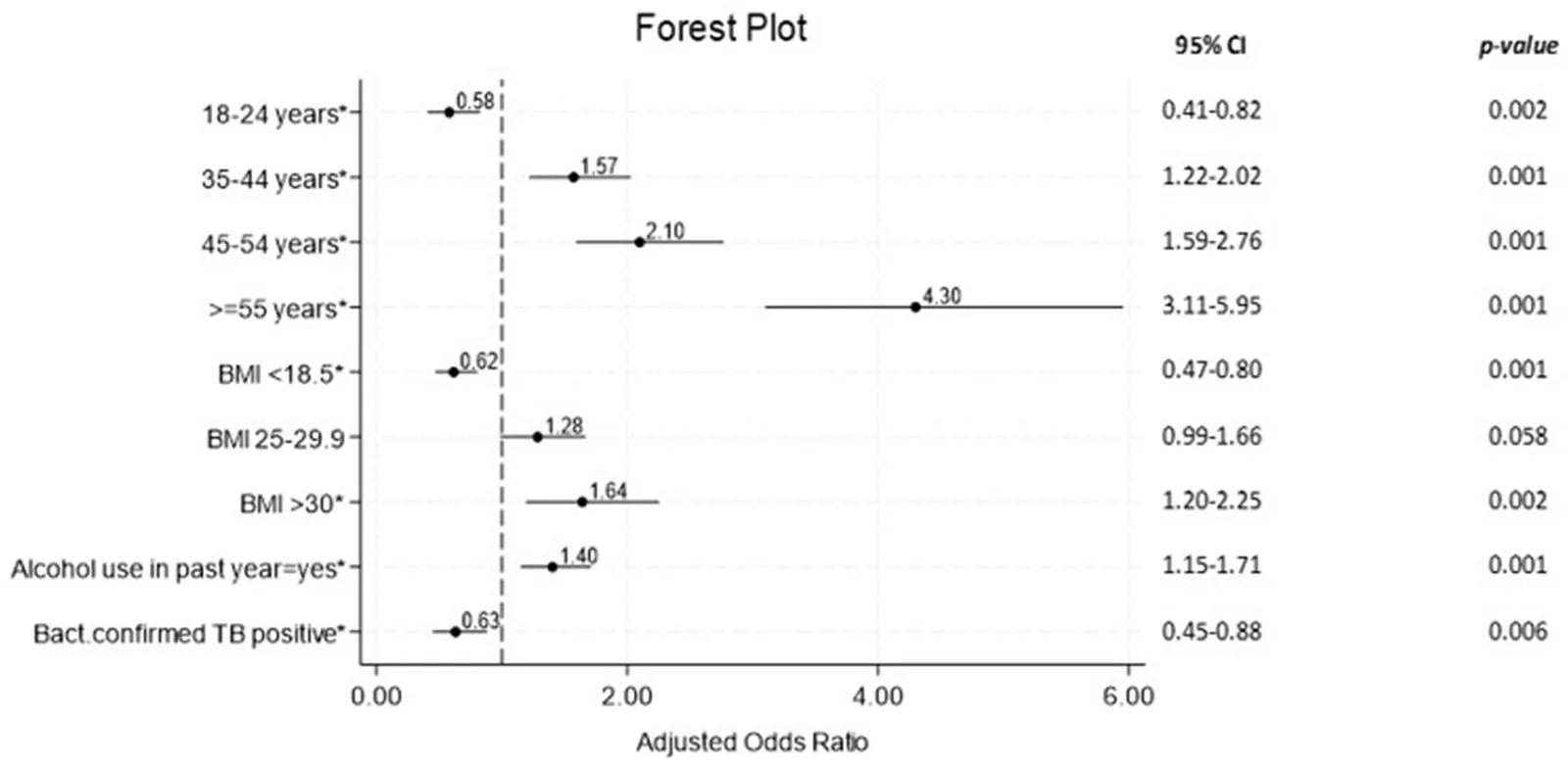
Forest plot for multivariable logistic regression of factors associated with HBP readings among persons with presumed and diagnosed TB (*N* = 2,431). TB, tuberculosis; Bact, Bacteriologically; BMI, Body Mass Index; *, Significant *p-value*; CI, Confidence Interval.

In the multivariable logistic regression analysis (Figure 2), after controlling for confounding variables, the odds of high BP were significantly higher in those older 34 years: 35–44 (aOR = 1.57, 95%CI = 1.22–2.02), 45–54 (aOR = 2.10, 95%CI = 1.59–2.76), and ≥55 (aOR = 4.30, 95%CI = 3.11–5.95). Expectedly, those who were obese had significantly high odds of high BP (aOR = 1.64, 95%CI = 1.20–2.25), while being overweight had insignificant odds for HBP (aOR = 1.28, 95%CI = 0.99–1.66). Lower odds of high BP readings were observed in individuals with bacteriologically confirmed TB (aOR = 0.63, 95%CI = 0.45–0.88; see Figure 2).

### Factors associated with treatment outcomes among TB patients

In the univariable logistic regression analysis (Table 2), among the factors analyzed, none demonstrated statistically significant associations with poor treatment outcomes, as indicated by the *p-values* exceeding 0.05. The majority of those diagnosed with TB had good treatment outcomes at 87.5% and we found no statistically significant association between HBP and poor treatment outcomes.

**Table 2:** Univariable logistic regression analysis of factors associated with treatment outcomes among bacteriologically confirmed TB patients (*N* = 287).

**Characteristics**	**Overall**	**Good treatment outcomes (251, 87.5%)**	**Poor treatment outcomes (36, 12.5%)**	**Univariable estimates**	
	***n*** **(%)**	***n*** **(%)**	***n*** **(%)**	**COR (95% CI)**	***p*** **-** ***value***
**Age group (years)**					
18–24	49 (17.1)	40 (15.9)	9 (25.0)	1.98 (0.75–5.25)	0.170
25–34	98 (34.2)	88 (35.1)	10 (27.8)	Ref.	Ref.
35–44	78 (27.2)	67 (26.7)	11 (30.6)	1.44 (0.58–3.60)	0.430
45–54	44 (15.3)	40 (15.9)	4 (11.1)	0.88 (0.26– 2.98)	0.837
>55	18 (6.3)	16 (6.4)	2 (5.6)	1.10 (0.22–5.50)	0.908
**Sex**					
Male	194 (67.6)	168 (66.9)	26 (72.2)	Ref.	Ref.
Female	93 (32.4)	83 (33.1)	10 (27.8)	0.78 (0.36–1.69)	0.527
**Patient type**					
Presumptive	285 (99.3)	250 (99.6)	35 (97.2)	Ref.	Ref.
Household contacts	0 (0.0)	0 (0.0)	0 (0.0)	–	–
HIV newly diagnosed/ < 1 month on ART	2 (0.7)	1 (0.4)	1 (2.8)	7.14 (0.44– 116.79)	0.168
**HIV status**					
Negative	180 (62.7)	159 (63.4)	21 (58.3)	Ref.	Ref.
Positive	107 (37.3)	92 (36.7)	15 (41.7)	1.23 (0.61–2.51)	0.561
**Prior TB**					
No	246 (85.7)	215 (85.7)	31 (86.1)	Ref.	Ref.
Yes	41 (14.3)	36 (14.3)	5 (13.9)	0.96 (0.35–2.64)	0.942
**Prior COVID-19**					
No	272 (94.8)	238 (94.8)	34 (94.4)	Ref.	Ref.
Yes	15 (5.2)	13 (5.2)	2 (5.6)	–	–
**BMI**					
< 18.5	121 (42.2)	107 (42.6)	14 (38.9)	0.72 (0.35–1.49)	0.380
18.5–24.9	137 (47.7)	116 (46.2)	21 (58.3)	Ref.	Ref.
25–29.9	20 (7.0)	20 (8.0)	0 (0.0)	–	–
>30	9 (3.1)	8 (3.2)	1 (2.8)	0.69 (0.11–0.29)	0.733
**Tobacco use**					
Never	151 (52.6)	130 (51.8)	21 (58.3)	Ref.	Ref.
Current	78 (27.2)	70 (27.9)	8 (22.2)	0.71 (0.30–1.68)	0.433
Stopped	58 (20.2)	51 (20.3)	7 (19.4)	0.85 (0.34–2.12)	0.727
**Alcohol use in past year**					
No	131 (45.6)	117 (46.6)	14 (38.9)	0.73 (0.36–1.49)	0.386
Yes	156 (54.4)	134 (53.4)	22 (61.1)	Ref.	Ref.
**Blood pressure**					
Normal (< 140 Sys, < 90 Dias)	237 (82.6)	211 (84.1)	26 (72.2)	Ref.	Ref.
High (≥140 Sys, ≥ 90 Dias)	50 (17.4)	40 (15.9)	10 (27.8)	2.03 (0.91– 4.53)	0.085

TB, tuberculosis; BMI, Body Mass Index; Sys, Systolic; Dias, Diastolic; >, greater than; <, less than; COR, Crude odds ratio; CI, Confidence interval.

## Discussion

We found that the prevalence of high blood pressure (HBP) among persons assessed for TB was considerably high at 1 in 4. High blood pressure was associated with increasing age, increasing BMI, and alcohol use in the previous year.

The prevalence of high blood pressure (HBP) among individuals assessed for TB in our study significantly exceeded the global estimate for hypertension in the general population as reported by the World Health Organization (WHO) at 1.28 billion out of 8.1 billion (~1 in 6 individuals) (2, 18). Our prevalence was similar to findings from a study conducted in India that compared the prevalence of hypertension between individuals diagnosed with TB and those without TB which reported that as high as a quarter of their patients had hypertension. However, they concluded that the burden of hypertension in those diagnosed with TB was no different from those without the disease in their population (19). Although no clear causality has been established in literature to explain the high prevalence of HBP among TB patients, some hypotheses suggest that it could be due to endothelial compromise arising from immunological triggers by *Mtb*. This could possibly increase the risk of cardiovascular diseases and hypertension. Another hypothesis relates to TB affecting renal parenchyma causing damage and resulting in impaired renal capacity to regulate blood pressure (14, 20, 21). Despite these prevailing hypotheses, we found a negative association between bacteriologically confirmed TB and HBP in our population.

The findings of a negative association between HBP and bacteriologically confirmed TB could have been driven by the impact of younger age and lower BMI which also had statistically significant lower odds of HBP in our study population (22). The finding may also be alternatively explained by vasodilation from a systemic inflammatory response or the loss in muscle mass or cachexia, both occurring in those with tuberculosis and possibly contributing to lower BP measurements (23). Other studies have reported that over 30% of individuals diagnosed with TB have adrenal insufficiency which could ably explain the lower BP among those with bacteriologically confirmed TB (24, 25). However, our study findings and the existing theories need to be further explored with larger and specifically designed studies for a more robust conclusion.

Studies on hypertension conducted in urban and rural areas of Zambia revealed similarly high proportions of individuals with HBP. Goma et al. (26) reported a prevalence of 38.4% whereas Tetayama et al. (27) found a prevalence of 39.7% and 33.5% in males and females, respectively. It is noteworthy that both studies used similar BP thresholds for defining HBP as our own study and also noted that BMI > 25, older age, and alcohol use were significantly associated with HBP (26, 27). Notwithstanding, none of these local studies compared the prevalence of HBP in the general population to that of persons assessed for or diagnosed with TB. A cross-sectional study of national electronic medical records among persons living with HIV in Zambia, disclosed a contrasting lower prevalence of HBP of 14.7% (28). The difference in prevalence's could be explained by the HIV study's focus on medical records of patients engaged in healthcare, which considered BP measurements taken during numerous interactions with health facilities. Whereas, the other studies only documented BP measurements among patients at first contact.

Persons with presumed and those at high risk for tuberculosis (TB) with a history of alcohol use were found to have an elevated risk HBP. This observation is particularly intriguing due to the established status of alcohol use disorder as an independent risk factor for TB, as well as a notable contributor to the incidence of cardiovascular diseases including hypertension. Several studies have similarly linked alcohol consumption to both TB and HBP, as highlighted by Imtiaz (29), Peng (30), and Fuchs (31). Additionally, Marak et al.'s (19) examination, comparing the prevalence of hypertension and alcohol consumption between TB patients and non-TB controls, also revealed higher rates of both conditions among individuals with TB. Concurrently, our research unveiled a heightened likelihood of HBP among obese participants being assessed for TB, while the opposite was true for underweight participants. This finding aligned with established connections between obesity and hypertension in the general populace (32–34). This was in contrast to the findings of Hsien et al. and others who reported an inverse association between obesity and risk of TB (35–38).

The coexistence of elevated BP levels with obesity or alcohol use in presumed TB patients has the potential to exacerbate the prognosis for individuals diagnosed with TB. While our study did not identify poorer treatment outcomes, it is plausible that disease severity may be more pronounced in individuals with comorbidities, although this aspect was regrettably not explored in our analysis. Our findings suggest that individuals undergoing TB assessment, particularly those with risk factors like alcohol consumption, older age and obesity, should undergo routine screening for HBP to effectively address potential comorbidities at an early stage in TB patients, a conclusion that aligns with the research findings of Baluku et al. (39).

For the assessment of treatment outcomes among those with bacteriologically confirmed TB, it was observed that individuals with HBP exhibited a higher proportion of poor outcomes with a ratio of 1 in 5, compared to those with normal BP whose ratio stood at about 1 in 9. This agreed with studies from Guinea-Bissau and Brazil that reported that TB patients with hypertension were unlikely to have good treatment outcomes (15, 40). However, in our study, we noted that univariable analysis of factors associated with treatment outcomes among participants diagnosed with TB, revealed no statistically significant association between HBP and poor treatment outcomes. This finding could have been due to the study design that was not primarily designed to assess outcomes of TB treatment and thereby lacked sufficient power to demonstrate this. The difference between our results and those from the cited studies may have arisen from the fact that our study participants had closer interactions with the study team in comparison to participants evaluated in the other studies that analyzed retrospective data from routine services.

One significant constraint of our research lay in the fact that most subjects had only one blood pressure measurement taken upon study enrollment. This proved insufficient for determining their baseline ambulatory and office blood pressure levels according to the diagnostic guidelines outlined by the International Society of Hypertension for global hypertension practice (17). Furthermore, our study was not specifically designed or adequately powered to investigate the correlation between high blood pressure and tuberculosis.

A major strength in our study is that it demonstrated the viability of incorporating non-communicable disease (NCD) screening within TB services. The dual impact of communicable diseases and NCDs presents a significant burden on individuals and healthcare systems. There exists a failure to seize opportunities for identifying comorbid conditions for coordinated treatment, which could result in both improved outcomes and health service cost savings. The implementation of bidirectional screening within TB clinics for early detection of HBP and other NCDs should be recommended to enhance patient care and provide comprehensive health services.

## Conclusion

The prevalence of high blood pressure (HBP) among persons assessed for TB was substantial. Factors associated with an increased risk for HBP included BMI over 30, age >35 years, and history of alcohol use. Consequently, our results provide valuable baseline data to inform strategies for integrated TB and non-communicable diseases (NCDs) including HBP screening in similar settings, paying particular attention to identifying and addressing risk factors associated with HBP among individuals presenting to TB services.

## Data Availability

The datasets presented in this study can be found in online repositories. The names of the repository/repositories and accession number(s) can be found in the article/Supplementary material.

## Supplementary material

The Supplementary Material for this article can be found online at: https://www.frontiersin.org/articles/10.3389/ftubr.2025.1548843/full#supplementary-material The datasets analyzed for this study can be found in the CIDRZ repository https://pubs.cidrz.org/handle/123456789/10815.
