# Supplementary material for: Prevalence and risk factors of high blood pressure among persons assessed for TB at three health facilities in Lusaka, Zambia
Source: Front Tuberc. 2025 Jul 9;3:1548843. doi: 10.3389/ftubr.2025.1548843 (PMC13491444; doi:10.3389/ftubr.2025.1548843)
Supplement: Supplementary file 1 [file Data_Sheet_1.pdf]

Supplementary Material

1 Supplementary Table

Table 3 (supplementary): Cross tabulation of high blood pressure and known hypertensive (N=2,431)

**Known Hypertensive**	**All**	**Blood Pressure Status**	
		High BP (n=653, 26.9%)	Normal BP (1,778, 73.1%)
Yes	265 (10.9)	175 (26.8)	90 (5.1)
No	2,166 (89.1)	478 (73.2)	1,688 (94.9)

Abbreviations: BP- Blood pressure
